# Characteristics of Aniline Aerofloat Biodegradation in Mineral Processing Wastewater and Energy Recovery by Single-Chamber Bioelectrochemical System: Strategies for Efficiency Improvement and Microbial Mechanisms

**DOI:** 10.3390/microorganisms13112610

**Published:** 2025-11-16

**Authors:** Xiaoyu Han, Wenchao Ji, Shengxiao Wang, Jingru Zhao, Hong Yu, Jiayang Ma, Meng Zhang, Jinyan Zhou, Xin Zhao

**Affiliations:** School of Resources and Civil Engineering, Northeastern University, Shenyang 110819, China; xiaoyuhan@mail.neu.edu.cn (X.H.); jiwenchao019@163.com (W.J.); wsxwsx202111@163.com (S.W.); 13070639097@163.com (J.Z.); 23s054058@stu.hit.edu.cn (H.Y.); 18041374726@163.com (J.M.); zhangmeng@mails.neu.edu.cn (M.Z.); zhoujinyan@mails.neu.edu.cn (J.Z.)

**Keywords:** mineral processing wastewater treatment, microbial fuel cell, aniline aerofloat, bacterial diversity, electricity production

## Abstract

Aniline aerofloat (AAF) is a typical refractory organic regent residual in mineral processing wastewater (MPW). Microbial fuel cells (MFCs) have been proven highly effective in degrading organic contaminants and resource recovering in wastewater treatment processes. However, AAF biodegradation potential and the related mechanisms in MFC systems remain poorly understood. In this study, the degradation of AAF, electricity generation performance and microbial mechanisms in the single-chamber MFC (sMFC) were confirmed. Affecting factors including AAF concentration, operation resistor, and pH were analyzed. The results indicated that under initial sodium acetate/AAF concentration of 300/100 mg/L, pH 7.0 and an operation resistor of 200 Ω, the AAF removal efficiency achieved 72.7 ± 1.6% with an output voltage of approximately 232 mV. The existence of AAF increased the relative abundance of electroactive bacteria, especially *Comamonas* and *Geobacter*. Functional prediction analysis showed that carbohydrate metabolism pathways was the dominant process. The relative abundance of N-respiration and S-respiration functional groups significantly increased, thereby improving COD and AAF removal. This study demonstrated that the MFC anode was beneficial to AAF degradation and provided an alternative route for the biodegradation of organic mineral processing reagents. To our knowledge, this is the first study evaluating AAF biodegradation performance in the MFC system.

## 1. Introduction

The wastewater from the non-ferrous metal mining industry occupies about 30% of the total discharged wastewater worldwide [1]. Mineral processing wastewater (MPW) contains wastewater from washing, comminution, beneficiation, and final tailings [2]. The main pollutants in MPW include suspended solids, heavy metal cations, and residual organic flotation reagents [3]. Flotation is a typical method for ore extraction, and one ton of raw ore needs 5–7 tons of water [4]. In China, the annual output of flotation wastewater is estimated to be 1.2 billion cubic meters and the majority of MPW is discharged into natural water bodies without treatment [5,6]. Excessive flotation reagents including collectors, frothers, and modifiers in wastewater have induced severe environmental problems and become a major challenge in wastewater management [7]. Aniline aerofloat (AAF, dianilinodithiophosphoric acid, (C_6_H_5_NH)_2_PSSH) is a widely used organic flotation collector in mineral processing. Around mine areas, the concentration of AAF has reached 0.45–1.43 mg/L in rivers and irrigation water [8]. AAF is hazardous and toxic to living beings and has negative effects on ecological environment, characterized by being hard to degrade and emitting unpleasant odor that poses risks to the environment [7,9]. Therefore, developing economical and efficient methods for the removal of AAF in mineral wastewater is of critical importance.

At present, various physical and chemical methods for treating MPW are extensively utilized, such as coagulation–flocculation (CF), chemical sedimentation, adsorption, advanced oxidation processes (AOPs) including the Fenton process, ozone oxidation, persulfate-advanced oxidation, etc. [4,10,11,12,13]. In the Fenton system with an Fe/Mn catalyst, more than 97% AAF was removed in 120 min [10]. Through the catalytic ozonation process, more than 90% AAF was removed in 180 min [14]. Hu et al. found that about 90% AAF was removed by oxidized pyrite activating persulfate [15]. However, most of these methods suffer from drawbacks of generating secondary pollution and substantial cost implications. As a cost-efficient and environmentally friendly approach, biological treatment can also be applied for organic flotation reagent biodegradation. Biological treatment has emerged as a promising alternative for MPW treatment, attributed to its cost-effective and environmentally friendly operation, reliable performance, and excellent effluent quality. Previous studies have indicated that AAF could be effectively removed in the bioreactors. In the submerged membrane bioreactor (sMBR), a removal rate of AAF up to 80% was achieved, and *Zoogloea*, *Clostridium*, *Sideroxydans lithotrophicus*, *Thiobacillus*, *Thauera amino aromatica*, and *Alicycliphilus denitrificans* were enriched in the sMBR [16]. Zhang et al. achieved 79% AAF degradation in the constructed rapid infiltration systems, the dominant bacteria were *Pseudomonadota* and *Actinomycetota* [17]. Ye et al. found that *Burkholderia* immobilized on biochar could degrade 80% of AAF within 72 h [8]. *Bacillus vallismortis* isolated from a sequencing batch reactor system (SBR) removed 91.8% AAF in 36 h [18]. Just a few studies have identified the biodegradation of AAF. However, with the global pressures of water resources and energy shortages, the technique of resources and energy recovery from wastewater has attracted much attention compared with conventional wastewater treatment processes primarily targeting pollutant removal [19].

Bioelectrochemical systems (BES) provided an option for alternative bioremediation technologies of hazardous pollutants. Microbial fuel cells (MFCs) are a novel bioelectrochemical technology that can convert chemical energy in organic substrates into electrical energy by electroactive microorganisms (EAMs) [20]. Various refractory organic pollutants can be degraded in MFC systems, such as toluene, phenol, aniline, polycyclic aromatic hydrocarbons (PAHs) [21,22,23,24,25]. The microbial community structure of electroactive biofilms plays a significant role in the degradation of refractory organics. As the extracellular electron transfer (EET) rate of electroactive biofilms is the determining factor for microbial overcoming of opening aromatic ring thermodynamic barriers [26]. The internal electric field of microorganisms of MFC provided additional “redox power” to the bacteria, enhancing the EET rate and accelerating the biotransformation of refractory compounds [27,28]. The MFC electrical performance and hazardous compounds degradation are also related to electrolyte conductivity and other operational factors. To date, the performance of MFCs for AAF degradation remains unrevealed. It is essential to clarify the effects of AAF on MFC systems, which will facilitate the development of high-efficiency biological treatment technology for the treatment of MPW.

In this study, the degradation potential of AAF, the influence factors including AAF concentration, solution pH, operating resistor and the microbial community of anode were investigated in the MFC. The degradation efficiencies and electricity output performance were analyzed. Bacterial community succession and the potential functional bacteria were investigated. The study aims to demonstrate the capability of the MFC bioanode for AAF degradation. The feasibility of AAF degradation in the MFC anode provides alternative suggestions for the AAF removal in mining industrial wastewater. To our knowledge, this is the first study to elaborate on the removal performance of AAF in MFC systems.

## 2. Materials and Methods

### 2.1. Reactor Construction

The single-chamber MFC was constructed with Plexiglas as described in the previous study [29]. The empty volume of the chamber was 28 mL (φ 3.7 cm, height 4.0 cm). The graphite brush (φ 3 cm × 3 cm) as anode was made by carbon fiber (ZOLTEK™ 3K, Toray Industries, Inc., Tokyo, Japan) with titanium wires. The anode was pretreated by soaking in acetone (100%) for 24 h and thermal treatment at 450 °C for 30 min. The air cathode, consisting of a gas diffusion layer, a catalyst layer and a stainless-steel mesh was prepared by the rolling method as described in the previous study [30]. The catalyst layer was made by activated carbon (Xinsen Carbon Co., Ltd., Nanping, China) and polytetrafluoroethylene (PTFE) (60 wt%, Hesen, Shanghai, China), the diffusion layer facing the air was constituted by carbon black (Jinqiushi Chemical Co., Ltd., Tianjin, China) and PTFE.

### 2.2. Inoculation and MFC Operation

**Figure 1:**
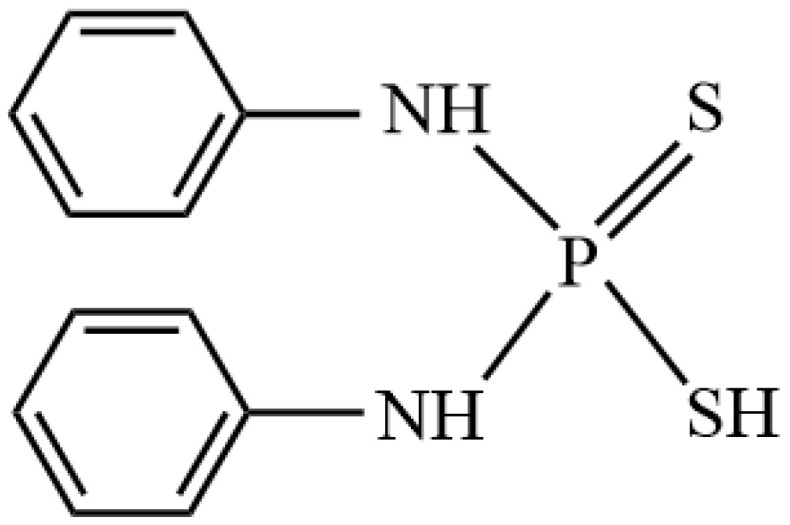
Molecular structure of aniline aerofloat.

The MFCs were inoculated with domestic wastewater from the Shenshuiwan Wastewater Treatment Plant in Shenyang, China. The growth medium at initial startup phase consisted of 200 mL/L domestic wastewater, 1 g/L CH_3_COONa (SA), and 50 mM/L phosphate buffer (Na_2_HPO_4_·12H_2_O 13.32 g/L, NaH_2_PO_4_·2H_2_O 3.32 g/L, and KCl 0.13 g/L). The MFCs generated stable currents after approximately 30 days of the inoculation process and AAF was added to the reactors. Industrial grade AAF was obtained from Wuhan Lullaby Pharmaceutical Chemical Co., Ltd. (Wuhan, China) (Figure 1). To investigate the AAF degradation efficiency in MFCs, different factors including SA/AAF mass ratio (300 mg/L SA + 50 mg/L AAF, 300 mg/L SA + 100 mg/L AAF, 100 mg/L SA+ 100 mg/L AAF), operating resistor (1000 Ω, 200 Ω, 50 Ω), and solution pH (5.0, 6.0, 7.0, 8.0) were explored to identify the effect of different factors (Table 1). The solution pH was adjusted by 1 mol/L HCl or 1 mol/L NaOH. All the experiments were operated at a constant temperature of 25 ± 2 °C.

### 2.3. Measurement and Analysis

AAF was quantified by the spectrophotometric method at 230 nm as the Supplementary Materials (Figures S1 and S2). Chemical oxygen demand (COD) concentration was measured by the method 5220 of HACH using a spectrophotometer (DR/3900 HACH Co., Loveland, CO, USA). The voltages of MFC reactors were recorded every 30 min by a data acquisition system (PISO-813, ICP DAS Co., Ltd., Hsinchu County, Taiwan). The polarization curve was obtained by changing the external resistor of MFC every 30 min. Before testing, the MFC was kept in open circuit state for 2 h. Power density was calculated according to P = UI/A, where U (V) is the output voltage, I is the calculated current by I = U/R, R (Ω) is the external resistance, and A (m^2^) is the surface area of cathode. The electrode potentials were measured with the reference electrode (Hg/Hg_2_Cl_2_ electrode, 0.280 V vs. standard hydrogen electrode, SHE). AC Impedance (EIS) was conducted on the workstation (CHI 660E, Chenhua Instruments, Shanghai, China). Columbic efficiency (CE) is the ratio of cumulative coulombs of a batch time and the theoretical coulombs calculated by COD consumption as Equation (1), where *t* is the reaction time, F is Faraday’s constant (96,485 C/mol e−), ΔC is the concentration variation of COD, V is the effective volume of influent, M is molecular weight of oxygen (16), and b is the number of electrons transferred by 1 mole O_2_, which is 4 [31].

(1) $$\mathrm{CE}=\frac{\int_{t=0}^{nt}Ut}{\mathrm{RFb}\Delta \mathrm{CV}}M$$

### 2.4. Bacteria Community Analyses

The bacterial community in the MFC anode was analyzed via pyrosequencing after one month of inoculation operation and at the end of the experiment. The bacterial 16S rDNA gene PCR was performed using 338F and 806R Primers targeting the variable region V3–V4. Pyrosequencing of amplicons was performed by Sangon Biotech Company (Shanghai, China) using the MiSeq instrument. PICRUSt2 was utilized to predict KEGG pathways and FAPROTAX was employed to analyze microbial function [32].

## 3. Results and Discussion

### 3.1. Performance of COD and AAF Removal in the sMFC

#### 3.1.1. Effect of Different SA/AAF Ratio and Operating Resistor

**Figure 2:**
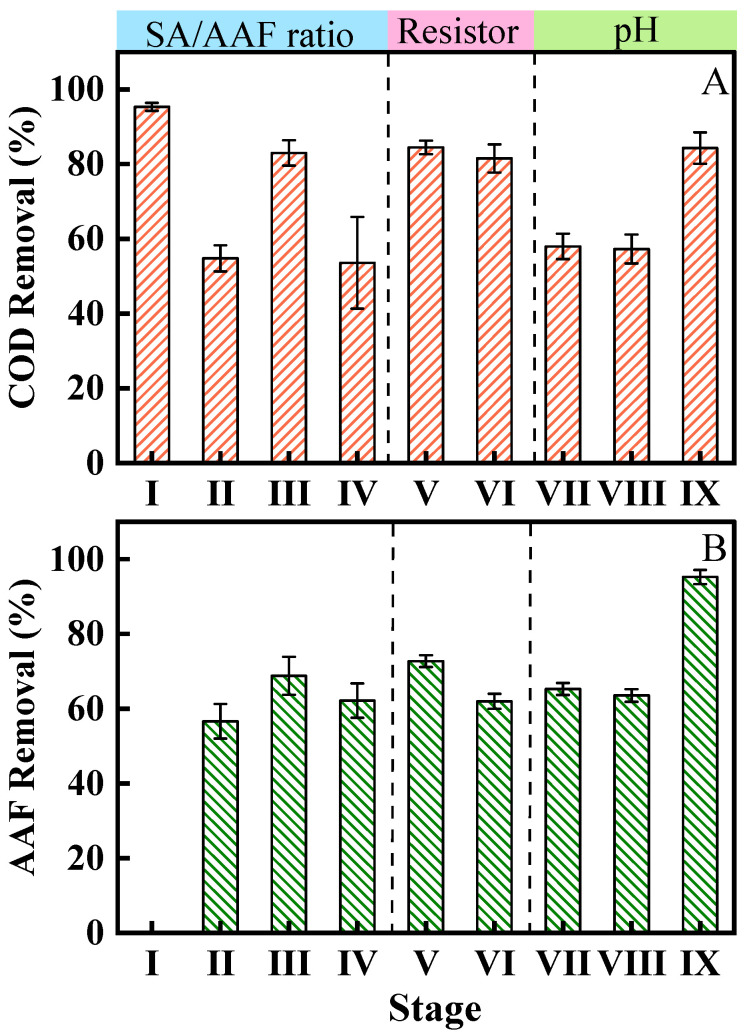
(**A**) COD removal and (**B**) AAF removal efficiency with different operation conditions.

To investigate the impact of different substrate concentrations on pollutant removal efficiency, the concentrations of SA and AAF were measured in the influent and effluent. 95.3 ± 1.1% COD removal was obtained with 1000 mg/L SA (Figure 2A). When the synthetic wastewater was 300 mg/L SA and 50 mg/L AAF, the removal efficiencies of COD and AAF achieved 54.8 ± 3.5% and 56.6 ± 4.6%, respectively (Figure 2B). After replacing with substances (300 mg/L SA and 100 mg/L AAF), the COD and AAF removal efficiencies increased to 83.0 ± 3.4% and 68.8 ± 5.1%. When SA concentration was reduced to 100 mg/L, the COD and AAF removal efficiencies approached 53.6 ± 12.3% and 62.2 ± 4.6%, respectively. The results showed that anode biofilm underwent an adaptation process, accompanied by a relatively low COD and AAF degradation efficiency [19]. The AAF degradation efficiency still improved when AAF concentration increased. With insufficient substrate available to the bacteria when SA was 100 mg/L, the AAF degradation decreased as the metabolic activity of bacteria was restricted.

When the sMFC was operated with different external resistances, the removal rates of COD and AAF were 84.5 ± 1.8% and 72.7 ± 1.6% with the resistance switched to 200 Ω (300 mg/L SA and 100 mg/L AAF). With 50 Ω resistance, the COD and AAF removal rates were 81.5 ± 3.8% and 62.0 ± 2.0%. The microbial electrochemical system exhibited the highest capability for AAF and COD removal when operated at 200 Ω. The MFC operation current increased with a smaller external resistor. Previous studies have reported that the operation resistance can impact the MFC anode biofilm activity [33]. The higher discharge current could enhance the electrochemical activities of biofilm. Maintaining a higher current requires a faster substrate consumption rate and stronger anode biofilm activity [34,35]. Consequently, reducing the external resistance increased COD consumption.

#### 3.1.2. Effect of Initial pH

**Figure 3:**
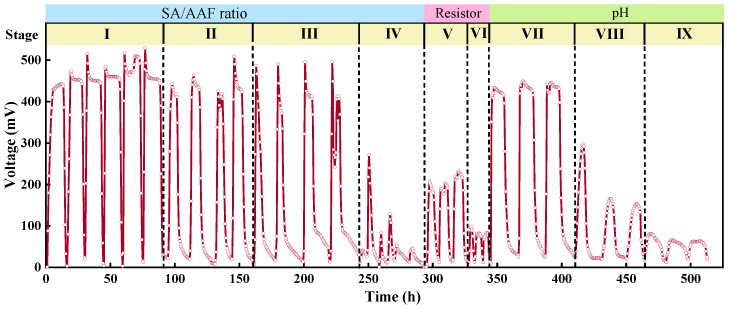
Output voltage of sMFC.

As in previous studies, electrolyte pH affects the MFC COD removal and electricity production performance [36]. The effect of electrolyte pH on pollutant removal is analyzed as Figure 2 Stage VII to IX. Compared with the performance observed at pH 7.0, the removal rates of COD and AAF decreased to 58.0 ± 3.4% and 65.3 ±1.6% at pH 8.0. At pH 6.0, they reached 57.3 ± 3.9% and 63.5 ± 1.7%. Conversely, COD and AAF removal rates were relatively high at pH 5.0, with average values of 84.3 ± 4.2% and 95.2 ± 1.9%, respectively. As AAF exhibits greater solubility in alkaline solutions, it likely tends to precipitate out of solution under pH 5.0, thereby leading to an overestimation of its removal efficiency. In conjunction with the results from Figure 3, the output voltage at pH 5.0 decreased nearly 80% compared with the voltage output at pH 7.0. This is due to the inhibitory effect of low pH on microbial activity [37]. Therefore, the higher removal rate should not be achieved by the microbial metabolic processes. Under weakly alkaline condition of pH 8.0 and acidic condition of pH 6.0, the decrease trend of removal of AAF and COD was presumably attributed to the inhibition of exoelectrogenic microbes and bacterial communities for AAF oxidation. Studies have demonstrated that extreme pH values adversely affect bacterial growth and metabolism at the anode, and pH 7.0 and 8.0 were the optimal pH for anode biofilm growth [36,38,39]. In this work, pH 7.0 was more favorable for high COD removal. Therefore, maintaining the electrolyte pH at 7.0 during operation can sustain the removal efficiency of COD and AAF in the sMFC.

### 3.2. sMFC Electricity Generation and Columbic Efficiency

#### 3.2.1. Electricity Generation Performance of sMFC

As shown in Figure 3, the output voltage fluctuated with the variation of synthetic wastewater, with 1000 mg/L SA the output voltage peak ranged from 454 mV to 510 mV, the maximum output voltage decreased to 509 mV–412 mV with 300 mg/L SA and 50 mg/L AAF. With the AAF increased to 100 mg/L, the maximum voltage decreased to 496 mV. The output voltage showed a sharp decrease (maximum voltage 217 mV~46 mV) when the influent changed to 100 mg/L SA and 100 mg/L AAF, which indicated that a lower SA concentration resulted in insufficient substrate available (280 mg/L COD) for microbial utilization and led to a decrease of microbial activity and electricity generation capacity. The sufficient COD from SA and AAF just induced a slight decrease of voltage output. The results indicated that MFC anodic biofilm could degrade AAF and generate electricity simultaneously. The existence of pollutant compound boosts the growth of the corresponding microorganisms specialized in metabolizing the compound [40]. As shown in the following microbial community analysis, the presence of AAF stimulated the enrichment of electroactive bacteria, which can also decompose aromatic compounds. EET is a complex process including various microbial mechanisms, previous investigations reported that some types of EET are more adaptive to the presence of contaminants [41,42].

**Figure 4:**
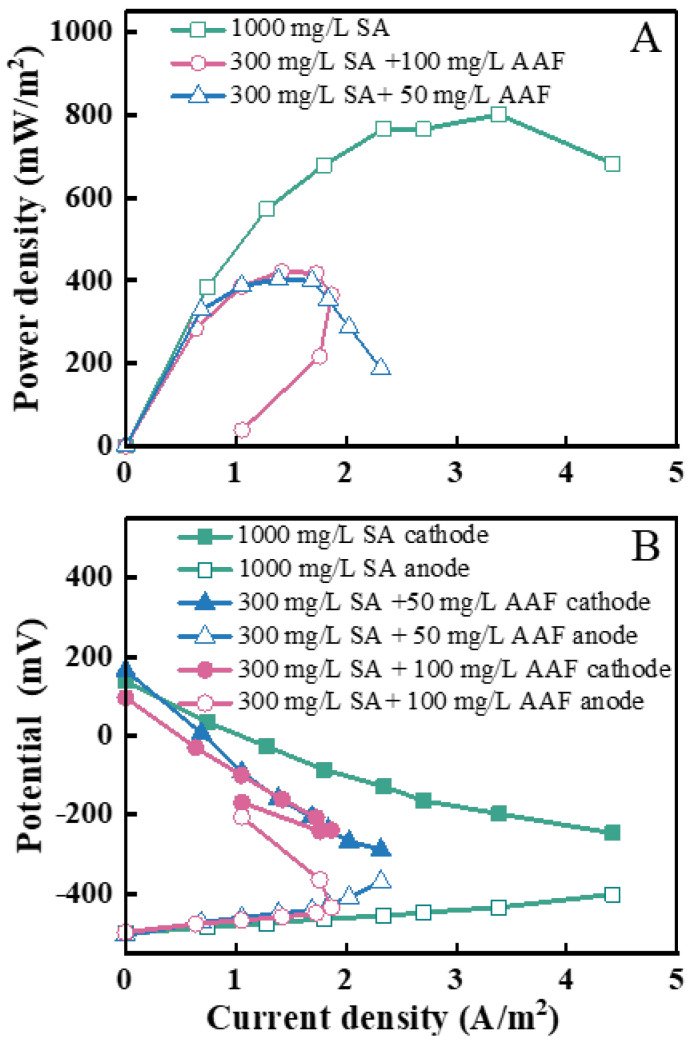
(**A**) Power density curves and (**B**) electrode polarization curves.

Polarization curves were measured to further analyze the electricity generation ability of the sMFC with sufficient substance (Figure 4A). With the SA/AAF ratio range, the maximum power densities of the MFC achieved 801.7 mW/m^2^, 422.1 mW/m^2^, and 402.6 mW/m^2^. The polarization curves indicated that COD concentration is the primary factor determining MFC maximum power density. Furthermore, with 100 mg/L AAF in the influent, a certain degree of power overshoot appears at the tail end of the polarization curves. This phenomenon, known as power overshoot, often occurs at high current densities beyond the maximum power point, where the cell voltage and current drop to lower values, resulting in a lower power output than before. Power overshoot is common in reactors adapted to high external resistance and often disappears when the anodic biofilm matures and produces sufficient redox enzymes. As shown in Figure 4B, the electrode polarization is greater for influents with lower COD. Polarization in the cell is a major cause of energy loss. The electrode potential deviation is greatest when the influent COD concentration is lowest, indicating severe polarization and increased energy loss. This also explains why both the MFC output voltage and maximum power density are lower under these conditions.

EIS was employed to investigate the distribution of the sMFC internal resistance in Figure S3. The ohmic resistances (R_ohm_) for the two influents were 27.9 Ω and 33.7 Ω, respectively, showing near-identical values. This is because the magnitude of ohmic resistance typically serves as a key factor determining MFC performance. In MFCs, R_ohm_ depends on reactor configuration rather than microbial characteristics on the anode electrode. The electron transfer resistance (R_ct_) depends on the conductivity of the reaction feed and contact resistance, while diffusion resistance (R_d_) depends on the diffusion rate of chemicals in electrolyte. Reactors with added AAF exhibited higher R_ct_ and R_d_ than those with single SA, indicating that AAF in the electrolyte increases the reactor’s internal resistance.

The external resistance significantly affects both the output voltage and the degradation of pollutants. When sMFC was operated with 200 Ω, the maximum output voltage was 232 mV. It reached approximately 90 mV with 50 Ω. When a lower external resistance was applied, the operation current increased. However, an excessively high current flow through the reactor can lead to electrode polarization, which adversely affects the MFC performance.

With the initial pH of influent adjusted from 8.0 to 5.0 for operation, the electricity generation capacity was inhibited by both acidic and alkaline conditions. The inhibition was relatively less severe at pH 8.0, with a maximum around 450 mV. In contrast, microbial activity was significantly inhibited at pH 6.0 and pH 5.0, resulting in a continuous decline in the discharge voltage to approximately 90 mV with pH 5.0. The results were consistent with previous studies, pH 7.0~8.0 is the optimal condition, and lower pH has adverse effects on MFCs [38].

#### 3.2.2. Coulombic Efficiency of the sMFC

**Figure 5:**
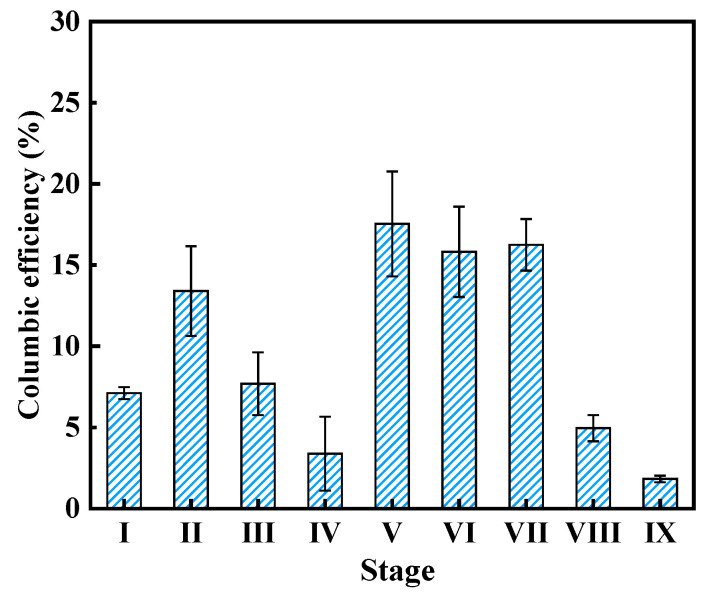
Coulombic efficiency of sMFC at different stages.

Coulombic efficiency (CE) is also one of the core indicators for evaluating the power generation performance of fuel cells. As illustrated in Figure 5, at different SA/AAF ratios (Stage I~IV), the CEs achieved 7.1 ± 0.4%, 13.4 ± 2.7%, 7.7 ± 1.9% and 3.4 ± 2.3%, respectively. Notably, the CEs at stage II and III with the addition of AAF were higher than stage I with sole SA substance, indicating that sMFC tended to generate higher electricity from a suitable ratio of SA and AAF. This phenomenon was consistent with the microbial community change, as a larger proportion of electroactive bacteria was enriched in the presence of AAF. With the decrease of operation resistor, CEs increased to 17.5 ± 3.2% and 15.8 ± 2.8%. With the variation of pH, the CEs were 16.3 ± 1.6%, 4.9 ± 0.8%, and 1.8 ± 0.2%, respectively, which were consistent with the trend of output voltage.

### 3.3. Microbial Community in the Anode Biofilm

#### 3.3.1. Microbial Community Analysis

The microbial community composition of the anode biofilms with different substrates was analyzed by 16S rRNA gene sequencing. As shown in Table S1, the number of operational taxonomic units (OTUs) increased from 503 (SA) to 546 (SA + AAF). The diversity indices, including Chao, ACE, and Shannon, demonstrated that AAF improved the microbial community richness and diversity.

**Figure 6:**
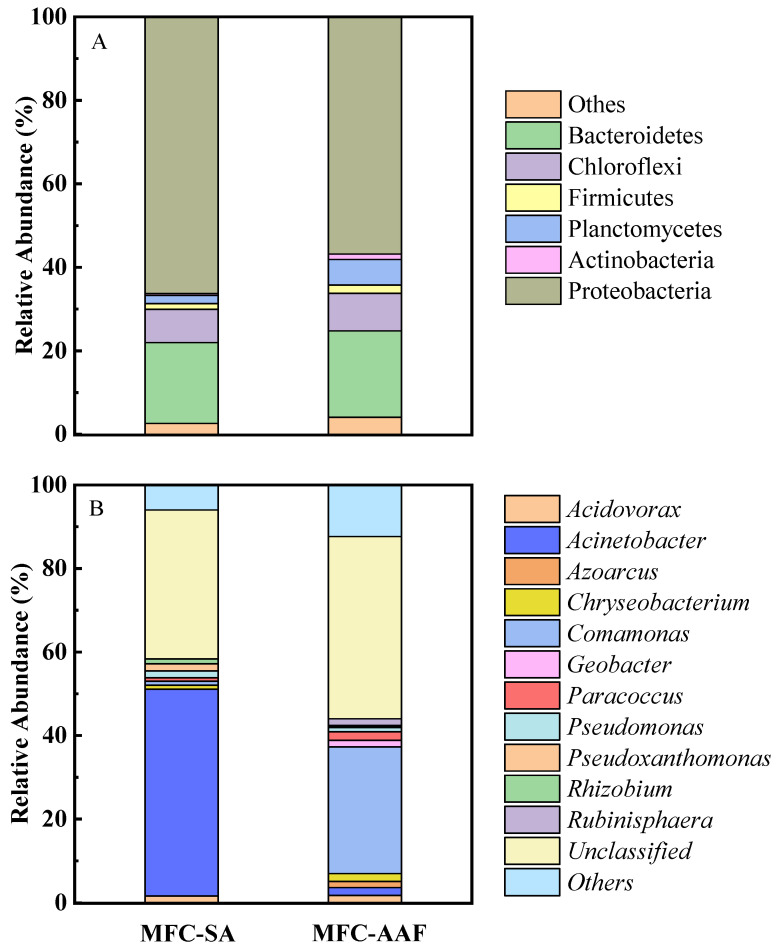
Relative abundance of microorganism at phylum level (**A**) and genus level (**B**) with different substance.

The sMFC anode showed different microbial community compositions with different substances at the phylum level (Figure 6A). The identified microbial phyla included Actinobacteria, Bacteroidetes, Chloroflexi, Firmicutes, Planctomycetes, and Proteobacteria. The dominant phyla were Bacteroidetes (19.4%) and Proteobacteria (74.0%) with SA substance and Bacteroidetes (20.7%), Proteobacteria (64.5%), and Planctomycetes (6.1%) with AAF addition. It is noteworthy that common electroactive microorganisms belong to the phyla Bacteroidetes, Proteobacteria, and Firmicutes. The relative abundance of Firmicutes phylum was significantly increased by adding AAF. Previous studies have indicated that microbes within this phylum could decompose complex organic compounds into simpler, more readily degradable substrates that facilitate electricity generation, suggesting the involvement in the AAF biodegradation.

The microbial community at the genus level was analyzed to demonstrate the AAF degradation and electricity production mechanism (Figure 6B). *Acinetobacter* (49.4%) was the dominant genus with the single SA substrate, while *Acidovorax* (1.7%), *Rhizobium* (1.2%) and the anode-respiring related genus *Pseudomonas* (1.6%) were also enriched. *Pseudomonas* has been identified as electrochemically active and capable of generating electricity by utilizing complex organic compounds as electron shuttles to facilitate electron transfer [43,44]. Multiple studies have demonstrated that *Pseudoxanthomas* is an exogenously electroactive bacterium. *Rhizobium* is a common nitrogen-fixing bacterium, no nitrogen source was added in the SA substance, *Rhizobium* is likely to provide a nitrogen source for the microbial community. *Rhizobium* can also directly capture H^+^ and electrons from the cathode as energy sources [45,46].

In comparison, with the cultivation of AAF, *Comamonas* (30.3%) was obviously enriched at the anode. The substantial enrichment of *Comamonas* at anode indicated the key role for AAF biodegradation. *Comamonas* is an electroactive genus and able to decompose aromatic compounds as the sole carbon and energy source anaerobically [20,47]. Furthermore, compared with anode with single SA, *Acinetobacter* (1.9%), *Pseudomonas* (1.0%), *Pseudoxanthomonas* (0.4%), and *Rhizobium* became less abundant when treating AAF. The genus *Acinetobacter* can degrade various aromatic compounds with multiple catabolic capacities [48]. *Paracoccus* (2.0%), *Chryseobacterium* (1.8%), *Acidovorax* (1.8%), *Rubinisphaera* (1.6%), and *Geobacter* (1.6%) were more abundant with AAF. *Paracoccus* is a common type of denitrification species found in bioelectrochemical denitrification systems and is also capable of producing electricity [49,50]. *Chryseobacterium* species have been industrially utilized for bioremediation of aromatic compounds, and this genus was found in anode biofilm for aromatic compounds treatment [51,52]. *Acidovorax* have both been reported to be electroactive and toluene-degrading bacteria [53]. *Geobacter* is a typical electroactive bacterial genus and has also been reported to degrade aromatic contaminants via oxidative ring cleavage [54].

The coexistence of various functional microbial communities in the anode biofilm supports the process of electricity generation and aromatic compounds degradation. AAF enhanced the enrichment of electroactive genera, including *Comamonas*, *Pseudomonas*, *Acidovorax*, and *Geobacter*. The dominant electroactive bacteria shifted from *Pseudomonas*, *Pseudoxanthomas*, and *Acidovorax* to *Comamonas*, *Acidovorax*, and *Geobacter*. Additionally, the enriched electroactive genera can degrade complex organic compounds to generate electricity. Notably, the coexistence of various functional microbial communities in the anode biofilm plays a pivotal role in sustaining electricity generation and AAF degradation.

#### 3.3.2. Microbial Function Predictive

**Figure 7:**
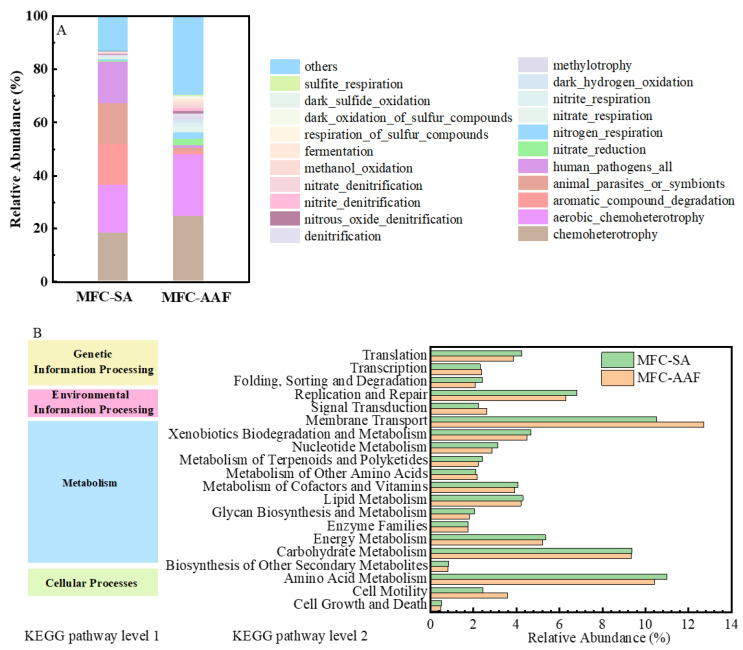
Predictive function pathways relative abundances (**A**), and potential functional bacteria (**B**).

To elucidate the metabolic mechanisms of microbial communities involved in in pollutant removal in the sMFC at different substrates, Kyoto Encyclopedia of Genes and Genomes (KEGG) was employed to predict the functional pathways associated with these bacteria (Figure 7A). Metabolism was the predominant functional category at different substrates, and amino acid metabolism, carbohydrate metabolism, energy metabolism, and xenobiotics biodegradation and metabolism had relatively higher abundances. Xenobiotics biodegradation and metabolism were linked to the degradation of organic substances, inferring the co-metabolic degradation of AAF and SA. However, the relative abundance of metabolic pathways with AAF decreased, which was likely due to the reduction of organic substances. The abundance of membrane transport, signal transduction, and cell motility increased with AAF, indicating the stronger interactions between bacteria and their surrounding environment, which were reflected in the key processes of material transport, energy exchange, information transmission, and EET. Cell motility is correlated with EET and cells also tended to migrate away from the toxic component. The above results suggest that AAF could be degraded and promote the EET process.

FAPROTAX prediction was employed to characterize the bacterial potential functions, as shown in Figure 7B. The chemoheterotrophy functional groups were the dominant (18.2% and 24.5%) [19,55]. The relative abundance of N-respiration and denitrification functional groups significantly increased from 3.0% to 12.9%, functional groups of S-respiration and oxidation increased from 0.1% to 1.7%, which indicated that the N and S elements in AAF were also transformed at the anode biofilm. In summary, the addition of AAF remarkably changed the bacterial structure to adapt to the pollutant in the system.

## 4. Conclusions

This study revealed the efficacy of sMFC in treating AAF in mineral processing wastewater. Higher substrate concentration promoted AAF degradation and electricity output, a smaller external operating resistor also improved the removal. Under operational conditions of 300 mg/L SA and 100 mg/L AAF, pH 7.0, and external resistor of 200 Ω, the maximum power density of sMFC achieved 422.1 mW/m^2^, with the removal rates of COD and AAF reaching 84.5 ± 1.8% and 72.7 ± 1.6%. Weak acidic and alkaline conditions led to a decline in electricity generation and AAF removal. The presence of AAF facilitated the enrichment of electroactive bacteria including *Comamonas*, *Pseudomonas*, *Acidovorax*, and *Geobacter*. *Comamonas* was the dominant genus in the anode biofilm. The sulfur and nitrogen respiration functional genera also helped to improve COD and AAF removal. MFCs can produce electricity from AAF and show great potential to recover power source from mining processing wastewater, which provide an alternative method for mining industry wastewater treatment.

## Figures and Tables

**Table 1:** Different operational stages of sMFC.

Stage	SA + AAF Concentration (mg/L)	Resistor (Ω)	pH
**I**	1000 + 0	1000	7.0
**II**	300 + 50	1000	7.0
**III**	300 + 100	1000	7.0
**IV**	100 + 100	1000	7.0
**V**	300 + 100	200	7.0
**VI**	300 + 100	50	7.0
**VII**	300 + 100	1000	8.0
**VIII**	300 + 100	1000	6.0
**IX**	300 + 100	1000	5.0

## Data Availability

The original contributions presented in this study are included in the article/Supplementary Materials. Further inquiries can be directed to the corresponding author.

## Supplementary Materials

The following supporting information can be downloaded at: https://www.mdpi.com/article/10.3390/microorganisms13112610/s1, Figure S1: The full-wavelength scan of AAF. Figure S2: Standard curve of AAF. Figure S3: EIS of sMFC with different substances. Table S1: The OTUs and diversity index of the sMFC anode with different substance.
