# Supplementary material for: Characteristics of Aniline Aerofloat Biodegradation in Mineral Processing Wastewater and Energy Recovery by Single-Chamber Bioelectrochemical System: Strategies for Efficiency Improvement and Microbial Mechanisms
Source: Microorganisms. 2025 Nov 16;13(11):2610. doi: 10.3390/microorganisms13112610 (PMC12654573; doi:10.3390/microorganisms13112610)
Supplement: Supplementary file 1 [file microorganisms-13-02610-s001.zip › microorganisms-3978950-supplementary.pdf]

Characteristics of Aniline Aerofloat Biodegradation in Mineral Processing Wastewater and Energy Recovery by Single-Chamber Bioelectrochemical System: Strategies for Efficiency Improvement and Microbial Mechanisms

Xiaoyu Han, Wenchao Ji, Shengxiao Wang, Jingru Zhao, Hong Yu, Jiayang Ma, Meng Zhang, Jinyan Zhou and Xin Zhao *

School of Resources and Civil Engineering, Northeastern University, Shenyang 110819, China

* Correspondence: zhaoxin@mail.neu.edu.cn

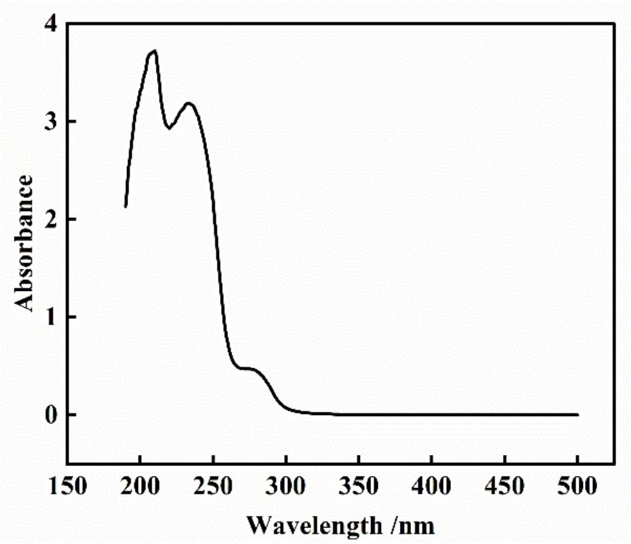

Fig.S1 The full-wavelength scan of AAF

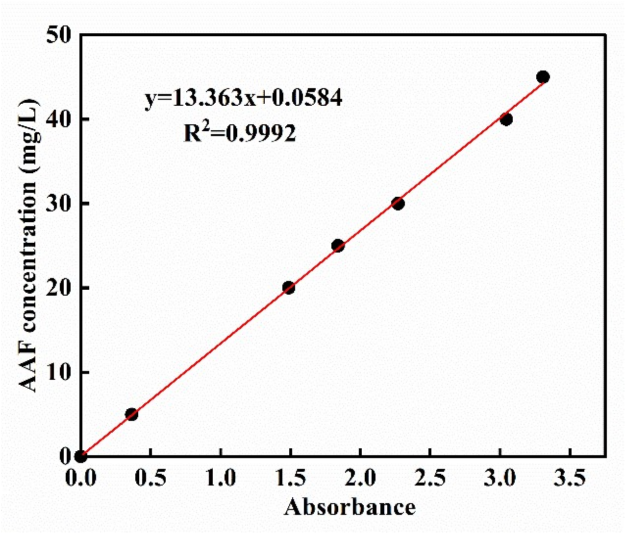

Fig. S2 Standard curve of AAF

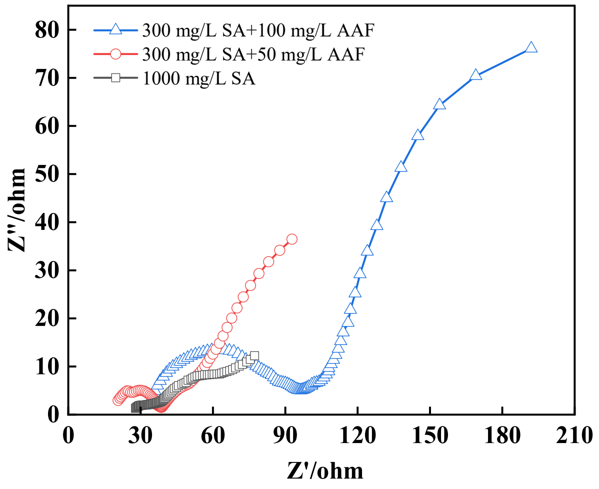

Fig.S3 EIS of sMFC with different substances

Table.S1 The OTUs and diversity index of the sMFC anode with different substance

Sample	OTUs	Shannon	Chao	Ace	Simpson	Shannoneven	Coverage
MFC-SA	503	2.98	506.18	516.96	0.1428	0.48	99.93%
MFC-AAF	546	3.5	549.77	560.16	0.1176	0.56	99.91%
